# Component Release and Mechanical Properties of Endodontic Sealers following Incorporation of Antimicrobial Agents

**DOI:** 10.1155/2017/2129807

**Published:** 2017-05-23

**Authors:** Elizabeta S. Gjorgievska, John W. Nicholson, Nichola J. Coleman, Samantha Booth, Aleksandar Dimkov, Andrew Hurt

**Affiliations:** ^1^Faculty of Dental Medicine, “Saints Cyril and Methodius” University of Skopje, Skopje, Macedonia; ^2^Bluefield Centre for Biomaterials, London, UK; ^3^Dental Materials Unit, Institute of Dentistry, Queen Mary University of London, London, UK; ^4^School of Science, University of Greenwich, Chatham, Kent, UK

## Abstract

Root canal sealers with antimicrobial activity are highly beneficial; therefore, their antimicrobial properties could be improved by incorporation of antimicrobial agents. In the present study, the release of the quaternary ammonium compounds from endodontic sealers admixed with either benzalkonium chloride (BC) or cetylpyridinium chloride (CPC) at loadings of 2% wt was monitored. The effect of these additives on the compressive strengths and their release from the sealers was determined after 1 and 4 weeks. All of the materials studied were found to be capable of releasing antimicrobial additive in useful quantities. The release of CPC occurred to a statistically significant greater extent than BC for all materials. The addition of both BC and CPC generally decreased the compressive strength of all the endodontic sealers, with the exception of CPC in AH Plus, where the compressive strength was significantly increased. This suggests that, for these endodontic sealers, the antimicrobial additives alter the setting chemistry. AH Plus is an epoxy-based material cured with an amine, and in this case the increase in compressive strength with CPC is attributed to an enhanced cure reaction with this system. In all other cases, the additive inhibited the cure reaction to a greater or lesser extent.

## 1. Introduction

The clinical practice of endodontics requires complete chemomechanical preparation and obturation of the root canals and postendodontic restoration in order to achieve optimal results in endodontic therapy. This is unquestionably of paramount importance in order to perform successful endodontic treatment.

The mouth hosts various species of potentially pathogenic bacteria, as a result of which infected root canals may contain many different microbial strains, mostly Gram-negative anaerobes [1–3]. In the study by Sundqvist et al. [4], the bacteria found in the root canal included* Enterococcus faecalis, Streptococcus anginosus, Bacteroides gracilis*, and* Fusobacterium nucleatum*.

Sealing generally involves the use of the semisolid material gutta-percha together with a sealing cement; the gutta-percha serves as the core-filling material, whereas the cement provides the seal to fill any spaces between the core-filling material and the dentinal walls [5].

With the currently available root filling materials, even a well obturated root canal is susceptible to reinfection by microorganisms. These can enter the system as a result of coronal leakage [6–9]. Hence, the root canal sealer is crucial in the prevention of bacterial leakage from the oral cavity along the gutta-percha and dentin interface into the periapical tissues [10]. In addition, the sealer is also more likely to come into direct contact with the remaining viable microorganisms in the dentinal tubules and any undebrided parts of the root canal system [11].

According to Grossman [12], one of the principal requirements of a sealer is that it should be bacteriostatic or at least not encourage bacterial growth. Ørstavik [13] confirmed that sealers play an important role in sealing the root canal system by the entombment of the remaining microorganisms and filling of inaccessible areas within the prepared canals. Consequently a sealer with antimicrobial activity is highly beneficial, because it can eliminate the remaining microorganisms present in the root canal after chemomechanical debridement and also prevent reinfection. Some root canal sealers are known to be inherently antimicrobial, a feature which can help to control the microorganism population [13]. However these antimicrobial properties are generally short-lived and rarely extend beyond 7 days [14]. In the clinical situation, this is insufficient to provide protection against persistent bacterial infection [15].

In principle, it is possible to improve the antimicrobial properties of dental materials by incorporating antimicrobial agents. For example, several antimicrobial substances have been used in mouthwashes, dental restorative materials, and toothpastes. These include chlorhexidine [16], cetylpyridinium chloride [17, 18], and benzalkonium chloride [17].

Cetylpyridinium chloride (CPC) is widely used as the active component of oral antiseptics and it is known to have broad spectrum antimicrobial properties, with a strong bactericidal effect on Gram-positive pathogens and yeasts in particular [18]. It is a quaternary ammonium compound which has a strong surface activity, and its effect on the reduction of plaque and calculus has been demonstrated previously [19]. In contact with the bacteria, CPC causes changes in the cell membrane, inhibition of the cellular functions, and cell death (bacteriolysis) [19].

The United States Pharmacopoeia recognises benzalkonium chloride (BC) as an auxiliary antimicrobial agent [19]. It is the major antimicrobial component in numerous toothpastes and mouthrinses, as well as in dental restoratives [20], and it is active against bacteria as well as certain viruses, fungi, and protozoa [21].

The objectives of the present study were to monitor the release of the quaternary ammonium compounds from four commercial endodontic sealers admixed with either BC or CPC at loadings of 2% by mass. The effect of these additives on the compressive strengths of the sealers was determined after 1 and 4 weeks. The null hypotheses tested were as follows: (a) there is no difference in the levels of quaternary ammonium compound released with or without added of antimicrobial agents at either 1 or 4 weeks; and (b) there is no difference in the compressive strengths of the sealers with and without antimicrobials at either time interval.

## 2. Materials and Methods

### 2.1. Sample Preparation

Four commercial endodontic sealers were used in this study as follows: Endomethasone N (Specialités-Septodont, Saint-Maur-des-Fossés, France); N2 (Hager & Werken GmbH & Co. KG, Germany); Apexit Plus (Ivoclar Vivadent AG, Schaan, Liechtenstein), and AH Plus (Dentsply-DeTrey GmbH, Konstanz, Germany). Full details of these materials are shown in Table 1.

Three sets of 12 samples (6 for each time interval) were prepared from each material, according to the procedure previously described by Markowitz et al. [22]: (a) without antimicrobial agent; (b) with 2 wt% CPC (*ex*. Sigma-Aldrich, UK); and (c) with 2 wt% BC (Fluka Chemical Corporation WI, USA). The samples without addition of an antimicrobial agent were prepared by mixing the components according to the manufacturers' instructions, while the samples with antimicrobial agents were prepared by mixing 2% by mass of the antimicrobial (CPC or BC, resp.) into the sealer. The prepared mixture was then placed in cylindrical metal molds of dimensions: 6 mm (height) by 4 mm (diameter). The molds were closed by clamped metal plates on both sides and incubated at 37°C for 24 h to set. After removal from the incubator and demolding, the specimens were stored individually in separate screw-capped polypropylene tubes in 5 cm^3^ of deionized water at room temperature for 1 or 4 weeks, when the testing took place.

### 2.2. BC and CPC Release

HPLC analysis was used to determine the concentrations of either the cetyl pyridium or the benzalkonium chloride released from the sealers at 1 and 4 weeks. The analysis was carried out using an Agilent 1200 system fitted with a reverse-phase C-18 Kromasil column of length 150 mm × 4.60 mm. A 20 *μ*l injection volume was used, and a flow rate of 1.0 cm^3^ min^−1^. The mobile phase was an isochratic system consisting of 55 : 45 acetonitrile : water with an added drop of glacial acetic acid. Detection used a variable wavelength detector set to 254 nm for BC and 259 nm for CPC. Standards of either BC or CPC were prepared at 25, 50, 100, 200, 300, and 400 ppm.

### 2.3. Compressive Strength

The compressive strength of the sealers was tested on six replicates of each sample-type according to the method described in ISO 9917-1:2007 [23] using a Universal Testing Machine (Instron Model 1193, Instron Corp., Canton, USA) at a crosshead speed of 1 mm min^−1^.

### 2.4. Statistical Analysis

The statistical analysis was performed by one-way analysis of variance (ANOVA) followed by post hoc Tukey's honest significant difference (HSD) test. The STATISTICA 7.1; SPSS 20.0 programme were used for data processing.

## 3. Results

The release of BC (Table 2) was highest from Endomethasone N (163.6 ppm and 459.8 ppm after weeks 1 and 4, resp.), while the lowest level of BC release was registered for Apexit Plus (28.3 ppm and 46.0 ppm after weeks 1 and 4, resp.). The extent of release of BC was significantly higher after week 4 for all of the tested materials.

After the first week, N2 had the highest level of CPC release (Table 3) with 98.0 ppm, although this was not significantly different from that of Endomethasone N, with 95.8 ppm. Again, the lowest level of CPC release was found for Apexit Plus (15.5 ppm and 34.2 ppm after weeks 1 and 4, resp.). The CPC release at week 4 was higher in all of the samples. The higher release of CPC compared with BC was statistically significant in all cases.

**Figure 1:**
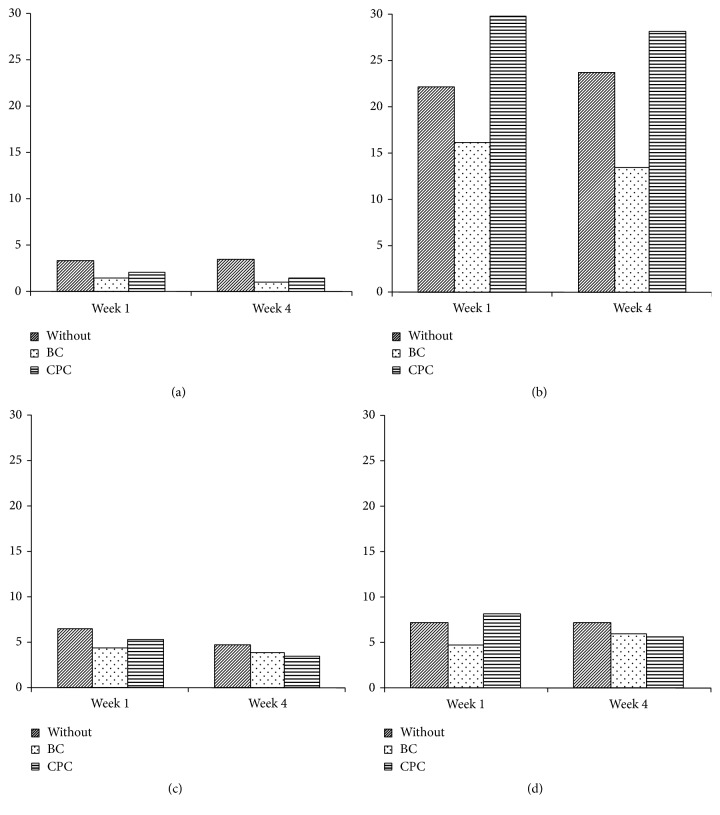
Compressive strength of endodontic sealers with and without addition of BC or CPC: (a) N2, (b) AH Plus, (c) Endomethasone N, and (d) Apexit Plus.

The highest compressive strength (Figure 1) was found for AH Plus, and the lowest was for N2. The addition of BC decreased the compressive strength of all of the endodontic sealers tested, while the addition of CPC decreased the strength of all materials, except for AH Plus, where the strength was significantly increased. A significant decrease in compressive strength was also noted between weeks 1 and 4 for Apexit Plus after the addition of CPC (*p* = 0.0260).

## 4. Discussion

The incorporation of antibacterial components into endodontic sealers may be a practical approach to preventing bacterial infection of treated root canals. Previous experimental studies have demonstrated that mechanical cleaning alone does not completely remove all bacteria [24, 25]. Once the canal is sealed, any possible reinfection will appear slowly, so that there is time for healing to occur. For persistent residual infection and coronal leakage, the use of an antimicrobial root canal sealer is highly desirable. Such a material would improve the chances of a successful treatment outcome. Inherent antimicrobial properties of materials are transient and rarely extend beyond 7 days [26]. This is generally not sufficient protection against persistent bacterial infection [22]. Hence, the use of antimicrobial additives in these sealants may be advantageous.

A previous study revealed that the addition of quaternary ammonium compounds (BC and CPC) increased the antimicrobial effect of the sealers, with significant increases in the zones of inhibition in all cases [24]. Results showed that the addition of 2% by mass of these antimicrobial compounds was sufficient to cause an increase in the antibacterial performance of the sealer in all cases. Even relatively inert materials, such as Roekoseal (Coltene Whaledent Ltd., UK), were found to be capable of being modified to have significant antimicrobial properties using this approach. Also, the bactericidal properties of inherently antimicrobial materials such as Endomethasone N and N2 were found to be enhanced by the addition of either BC or CPC at the mixing stage [18]. In endodontics, it is generally accepted that periapical inflammation is the result of bacterial infection [21], so that these modified materials have the potential to prevent such inflammation when used clinically.

In a previous study, BC was added to a chemically cured composite resin and antimicrobial activity increased with higher BC content. There were no significant differences in either tensile bond strength or diametral tensile stress among the modified composite groups and the original product. Therefore, the incorporation of BC in the composite material added antimicrobial properties to the original compound without altering its mechanical properties [23]. When AH Plus was tested alone and mixed with 1%, 2%, and 3% of BC, the physical properties of the sealers were according to the ANSI/ADA specifications; but, the microhardness decreased significantly when BC was added, and a significant reduction in contact angle was obtained when incorporating 2% and 3% BC (*p* < 0.05). It is obvious that the additives to the root canal sealer altered other physical and chemical properties that are not commonly found in the literature to evaluate filling materials and the authors suggested additional testing [27]. A decrease in mechanical strength was also found in another study which dealt with incorporation of antimicrobials in glass-ionomer cements, but it was stated that the microhardness decrease was tolerable, having in mind the improvements in the antimicrobial properties [28].

In general, our results showed that adding BC or CPC caused the compressive strength of the materials to fall. This leads us to reject null hypothesis (b) concerning compressive strength values. The exception to this was AH Plus with CPC, where compressive strength increased significantly. In the case of the chelate type materials, this reduction in strength indicates that the additives interfere with the setting chemistry, at least to an extent, as has been shown in similar studies using glass-ionomer cements [17, 21, 26, 29]. In the case of AH Plus, which is based on an epoxy system, the fact that the presence of the quaternary ammonium compound CPC improves the strength may be considered to arise from an enhancement of the setting process. Epoxies are typically cured by the use of amines, so that a quaternary ammonium compound is likely to be beneficial. Despite this, similar effects were not observed when BC was used as the antimicrobial additive in this material.

The determination of the compressive strength was employed in this study as a means of assessing the extent to which the various materials were altered by the addition of either BC or CPC. Compressive strength is not an important property of endodontic materials, as indicated by the wide variation in compressive strengths for the materials in this study, all of which are successful as endodontic sealers in clinical application. More important is the ability of these materials to adapt to the walls of the prepared root and thereby reduce or prevent bacterial contamination of the endodontically prepared tooth.

The release of the quaternary ammonium ions (BC and CPC) was shown by our results to continue between 1 and 4 weeks for all of the blended samples. The release of the antimicrobial agent was higher to a statistically significant extent from the samples containing CPC than from samples containing BC. This suggests that CPC is easier to be released, possibly because it is a smaller, less rigid molecule and therefore able to diffuse more readily through a variety of hardened materials. More important is the observation that all materials are able to release reasonable amounts of either BC or CPC, depending on formulation. The fact that the release of these substances occurs to the extents observed leads us to reject null hypothesis (a).

## 5. Conclusions

All of the materials studied were found to be capable of releasing antimicrobial additive in useful quantities, following their incorporation at the mixing stage. The release of CPC occurred to a statistically significant greater extent than BC for all materials.

The addition of both BC and CPC generally decreased the compressive strength of all the endodontic sealers, with the exception of CPC in AH Plus, where the compressive strength was significantly increased. This suggests that, for these endodontic sealers, the antimicrobial additives alter the setting chemistry. AH Plus is an epoxy-based material cured with an amine, and in this case the increase in compressive strength with CPC is attributed to an enhanced cure reaction with this system. In all other cases, the additive inhibited the cure reaction to a greater or lesser extent.

Our results lead us to reject both null hypotheses and to conclude that the presence of either BC or CPC causes both a change in the compressive strength of the endodontic materials studied and an increase in the release of antimicrobial compound. We conclude that the addition of these substances to endodontic sealers has potential to maintain a sterile root canal in clinical endodontics.

## Figures and Tables

**Table 1:** Endodontic sealers used in the study.

Sealer	Manufacturer	Composition
N2	Hager & Werken GmbH & Co. KG, Germany	*Powder*. Zinc oxide, zinc stearate, dehydrated zinc acetate, paraformaldehyde, titanium dioxide, basic bismuth subcarbonate, basic bismuth nitrate, yellow ferrous oxide *Liquid*. Eugenol, rose oil, lavender oil, peanut oil

AH Plus	Dentsply De Trey GmbH, Germany	*Epoxide Paste*. Diepoxide, calcium tungstate, zirconium oxide, aerosil, pigment *Amine Paste*. 1-Adamantane amine, N,N′-dibenzyl-5-oxa-nonandiamine-1,9, TCD-diamine, calcium tungstate, zirconium oxide, aerosil, silicone oil

Endomethasone N	Septodont Inc., France	Hydrocortisone acetate, thymol iodide, barium sulfate, zinc oxide, magnesium stearate

Apexit Plus	Ivoclar Vivadent AG, Switzerland	*Base*. Calcium hydroxide/calcium oxide, hydrated colophonium, fillers and other auxiliary materials (highly dispersed silicon dioxide, phosphoric acid alkyl ester) *Activator*. Disalicylate, bismuth hydroxide/bismuth carbonate, fillers and other auxiliary materials (highly dispersed silicon dioxide, phosphoric acid alkyl ester)

**Table 2:** Benzalkonium chloride release (ppm) from endodontic sealers.

Endodontic sealer	Week 1	Week 4	*p* value
	Concentration (SD) [ppm]	Concentration (SD) [ppm]	
N2	41.5 (1.2)	103.2 (7.3)	0.0000^*∗*^
AH Plus	47.9 (3.1)	126.7 (9.8)	0.0000^*∗*^
Endomethasone N	163.6 (8.0)	459.8 (17.6)	0.0004^*∗*^
Apexit Plus	28.3 (1.8)	46.0 (2.0)	0.0001^*∗*^

^*∗*^Statistically significant difference between week 1 and week 4 (one-way ANOVA followed by post hoc Tukey's honest significant difference (HSD) test).

**Table 3:** Cetylpyridinium chloride release (ppm) from endodontic sealers.

Endodontic sealer	Week 1	Week 4	*p* value
	Concentration (SD) [ppm]	Concentration (SD) [ppm]	
N2	98.0 (55.7)	142.4 (142.0)	0.0061^*∗*^
AH Plus	65.2 (56.9)	140.4 (106.7)	0.0000^*∗*^
Endomethasone N	95.8 (47.5)	152.0 (38.9)	0.0006^*∗*^
Apexit Plus	15.5 (13.0)	34.2 (22.1)	0.0000^*∗*^

^*∗*^Statistically significant difference between week 1 and week 4 (one-way ANOVA followed by post hoc Tukey's honest significant difference HSD test).
